# Discovery and validation of FBLN1 and ANT3 as potential biomarkers for early detection of cervical cancer

**DOI:** 10.1186/s12935-021-01802-5

**Published:** 2021-02-18

**Authors:** Yi Hao, Ming Ye, Xiaona Chen, Hongli Zhao, Ayshamgul Hasim, Xia Guo

**Affiliations:** 1 Department of Ultrasound, Shenzhen University Pinghu Hospital, Shenzhen University, Shenzhen, PR China; 2 Department of Pathology, Affiliated Cancer Hospital, Xinjiang Medical University, Urumqi, PR China; 3 Center for Clinical Research and Innovation (CCRI), Shenzhen Hospital, Southern Medical University, Shenzhen, PR China; 4 Department of Pathology, Basic College, Xinjiang Medical University, Urumqi, PR China

**Keywords:** HPV infection, FBLN1, ANT3, Serum tumor markers, Cervical carcinoma

## Abstract

**Background:**

To validate markers for cervical carcinoma (CC) and precancerous lesions related with HPV infections.

**Methods:**

Three different cervical cancer cell lines C-33A, SiHa and Caski were used for secretome profiling by label-free quantitative proteomics. Cervical exfoliated cells and matching serum samples were collected from 284 patients with normal control (n = 75, 26.41 %), precancerous lesions (n = 88, 30.99 %) and early stage cervical squamous carcinoma (n = 121, 42.61 %). HPV subtyping and quantification was performed by PCR and hybridization. 20 candidate proteins identified in previous screening studies (tissue, plasma, cells) were quantified by ELISA. Finally, highly quantitative parallel reaction monitoring mass spectrometry was used to assess the specificities and sensitivities of candidate serum markers.

**Results:**

While CC was found to be associated with high-risk HPV subtypes, serum antibodies for high risk HPV were not significantly related to the progression of cervical cancer. Significant differences between patient groups were detected for the four proteins CLU, APOA4, APOE and MLH3, but none would allow clinical application due to insufficient sensitivity and specificity and large variability. Subsequent proteomic secretome analysis of cervical cancer cell lines identified a set of 729 common proteins. Cross referencing this dataset with ELISA measurements revealed six candidate proteins of which two, FBLN1 and ANT3, showed co-occurrence with HPV infection (75.7 % and 85 %, respectively) and had promising diagnostic ability in terms of sensitivity and specificity. After the loss of E6/E7 by using CRISPR/Cas9 gene editing, the content of ANT3 and FBLN1 in KoE6/E7 SiHa were downregulated, which indicated the expression of ANT3 and FBLN1 in cervical cancer may be affected by HPV infection.

**Conclusions:**

FBLN1 and ANT3 might be potential tumor- and HPV-associated serum markers.

**Supplementary Information:**

The online version contains supplementary material available at 10.1186/s12935-021-01802-5.

## Background

Cervical carcinoma is one of the most common gynecological malignancies causing about 270,000 deaths per year worldwide [1]. In China, there are approximately 130,000 new cases annually, resulting in 50,000 deaths [2]. World Health Organization (WHO) statistics estimate that one new case is diagnosed every four minutes and about eighty women die from the disease every day in China [3]. Uyghur is a Turkic ethnic population mostly living in Xinjiang Autonomous Region, and the total population in China has exceeded 10 million [4]. Epidemiological surveys show that Uyghur women have a higher incidence and mortality of cervical cancer than Han women and other ethnic groups in the same area in recent years [5].

According to Surveillance, Epidemiology, and End Result program (SEER) analysis of death rates in the United States, for cervical cancer, 16 % are diagnosed when the cancer has metastasized and 5-year relative survival is 16.8 % [6]. Late stage cancer still has a poor prognosis although treatment options have improved (https://www.ncbi.nlm.nih.gov/books/NBK65801/). Therefore, regular screening is critical for effective early detection and treatment of cervical cancer [7, 8]. In current clinical practice in China, screening rests on morphological changes in cervical liquid-based cytology, and patients with abnormal test results undergo colposcopic cervical biopsy, depending on their HPV infection status [9, 10]. In addition, although resection is the preferred method of treating cervical carcinoma and precancerous cervical intraepithelial neoplasia, the procedure may cause infertility [11]. To date, there is still no simple and rapid test for early diagnosis and risk evaluation of HPV infection, cervical carcinoma, or precancerous lesions. Patients may, however, display changes in the levels of functional molecules or genetic mutations that could be used as biomarkers. Therefore, in the present study, we analyzed a large collection of serum samples with the aim of identifying diagnostic and prognostic molecular biomarkers for cervical diseases that might be applicable to patients in the Uygur-Han mixed region.

HPV infection is closely linked to the etiology of cervical precancerous lesions. Dysfunction of the cellular immune response is thought to underlie HPV persistence and subsequent cancer development [12]. Sensitive high-throughput methods are available to detect HPV DNA [13], which have become routine diagnostics worldwide. Furthermore, yet unknown serum biomarkers may exist that reflect the degree of HPV infection and HPV subtype with the potential to serve as indicators in early detection and diagnosis.

In our previous studies, we screened the serum proteome of patients with cervical lesions by 2-dimentional high performance liquid chromatography and mass spectrometry. A total of 103 differential serum proteins were identified in cervical carcinoma patients, among which 31 were found in patients with high-grade squamous intraepithelial lesions (HSIL) (Data not published). We also identified 16 differential proteins using 2-dimensional difference gel electrophoresis (2D-DIGE) combined with Matrix-Assisted Laser Desorption/Ionization Time of Flight Mass Spectrometry (MALDI-TOF-MS) and identified cervical carcinoma-related regulatory pathways. Candidate marker proteins were identified using Ingenuity Pathway and Meta Core™ Analysis platforms for the functional annotation and enrichment of differential proteins, establishment of related regulatory and signaling networks, and identification of potential molecular biomarkers for screening and evaluation of cervical carcinoma [14]. Based on integrating the above datasets, we focus on 46 proteins as potential markers for cervical cancer and precancerous lesions. These candidate indicators may become valuable biomarkers in the early clinical diagnosis of cervical carcinoma.

Women are vulnerable to cervical diseases including normal control, HSIL and early stage cervical carcinoma [15]. In the present study, specimens of cervical lesions were taken from Uygur patients as well as Han patients living in Xinjiang province. Enzyme-linked immunosorbent assay (ELISA) and parallel reaction monitoring mass spectrometry (PRM-MS) were used to qualitatively and quantitatively analyze the candidate differential proteins identified in our previous studies and to link them with HPV status. The integration of HPV status with candidate serum markers is expected to efficiently narrow down the high risk population and provide sufficient clinical information for early diagnosis and effective therapy.

## Methods

### Collection of clinical specimens

**Fig. 1:**
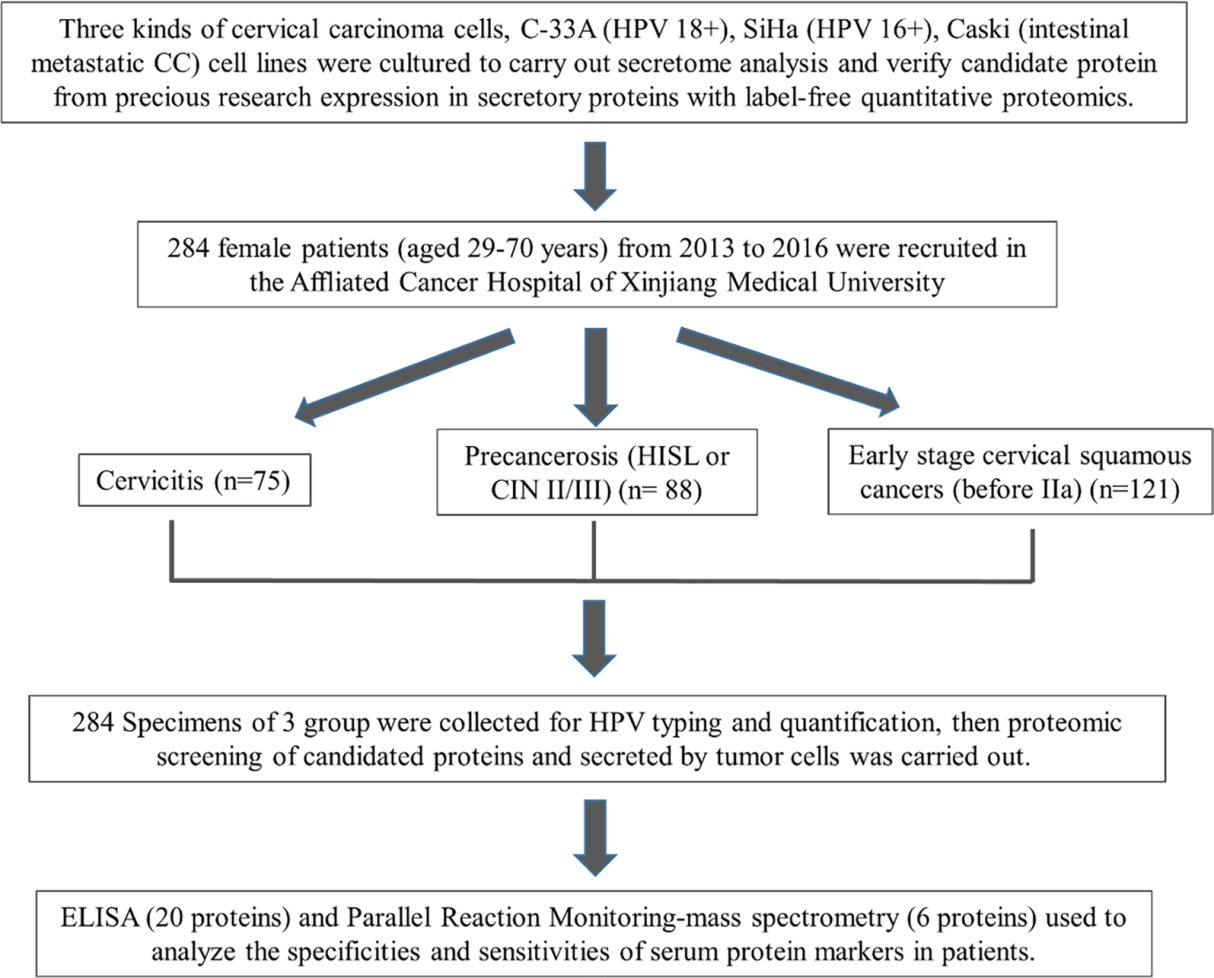
Flowchart of the study

Exfoliated cells from the cervix and blood plasma were collected from a total of 284 patients (aged 29–70 years, mean 48 ± 6.5) treated at the Affiliated Cancer Hospital of Xinjiang Medical University between 2013 and 2016. Specimens were from 75 cases of normal control, 88 cases with precancerous lesions (HISL or CIN II/III) and 121 early stage cervical squamous carcinomas (before IIa) (Fig. 1). Nearly equal numbers of cases were from Han and Uygur patients with a Han/Uygur ratio of 0.83:1. In line with World Health Organization recommendations (WHO, 1990 Version), the clinical diagnosis was made by a physician, followed by staging and subtyping of cervical lesions by a trained pathologist. Clinical data including medical histories were recorded. The study was reviewed and approved by the Ethics Committee of the Affiliated Cancer Hospital of Xinjiang Medical University, and written informed consent was obtained from each participant (G-201,442). The mass spectrometry proteomics data have been deposited to the ProteomeXchange Consortium via the PRIDE [16] partner repository with the dataset identifier PXD022756.

### Sampling of cervical exfoliated cells

2 days prior to specimen collection, subjects were asked to abstain from vaginal douching and sexual intercourse. Cervical exfoliated cells were collected using a sampling brush which was turned counterclockwise 3 times. Motion stopped for 10 sec at the cervix orifice, followed by immersing the brush into a tube containing cell preservation fluid. The specimen was processed and stored at − 80 °C until required.

Using standardized phlebotomy procedures, 10 mL of peripheral blood was drawn from each subject. Within 2 to 4 h of collection, blood samples were processed using guidelines recommended by the US National Cancer Institute Intergroup Specimen Banking Committee. Samples were stored at − 80 °C in the Biological Specimen Bank of the Institute for Cancer Research at the Affiliated Cancer Hospital of Xinjiang Medical University.

### Patient stratification

Cervical intraepithelial neoplasia (CIN) is widely seen as a reversible pathophysiological process. Most CIN I regress without treatment and only few patients develop CIN II or III. Concurrence of CIN II or III with carcinoma *in situ* is referred to as high-grade squamous intraepithelial lesion (HSIL), which has a high risk of progression to carcinoma. Therefore, we excluded patients with CIN I and combined patients with CIN II and III into the same group to minimize diagnostic and examination errors. Patients with cervical carcinoma stage < IIa and without metastasis were classified as early-stage. The same stratification criteria were applied for the early screening study to identify candidate proteins with differential abundance.

### HPV genotyping of cervical exfoliated cells

HPV genotyping has been basically performed according to a previous study [17]. A nucleic acid amplification and genotyping system (Hybribio, Hong Kong) was used for HPV detection and genotyping. Cells of a 500-µL sample were pelleted by centrifugation for 1 min at 14,000 rpm and DNA was extracted. 1 µL of DNA template was added to 24 µL pre-mixed PCR reaction containing Taq DNA polymerase for a final reaction volume of 25 µL. Positive (HPV-18) and negative controls were included in each amplification reaction. The Hybrimax kit (Hybribio, Hong Kong) was used for rapid flow-through hybridization of nucleic acid. Briefly, a Hybond membrane containing 37 oligonucleotide probes of each HPV genotype was positioned to perform hybridization and enzymatic detection according to the manufacturer’s instruction. A visible blue/purple dot was a positive signal and the HPV genotype was determined by the distribution map of the HPV genotype on the membrane. A total of 37 HPV genotypes including 16, 18, 31, 33, 35, 39, 45, 51, 52, 56, 58, 59, 66, 68, 6, 11, 42, 43, 44, 81, 53, 26, 34, 40, 54, 55, 57, 61, 67, 69, 70, 71, 72, 73, 82, 83 and 84, and multiple infections could accurately be detected with both sensitivity and specificity of > 95 %.

### Serum HPV antibody detection

A high risk Papilloma Virus L1-Capsids (HR-HPVL1) (IgG) ELISA kit (ABIN1000221, Antibodies-Online, US) was used to measure serum levels of HPV antibodies according to the manufacturer’s protocol. Briefly, duplicate readings for two standards and samples were averaged and the average blank reading was subtracted. Standard curves were fitted by 4-parameter logistic regression. Alternative, a standard curve was derived by linear regression of log antigen concentrations plotted versus the log OD readings. This procedure produced an adequate but less precise fit of the data. If samples were diluted, the derived concentrations were multiplied by the dilution factor.

#### ELISA

ELISA detection was performed to quantify 20 candidate proteins in plasma, which included: BIRC6, DCD, SPTBN4, HLA-DQB1, FBLN1, ANT3 (ADP/ATP translocase 3), VIL1, SERPINC1, IGK, ADCY2, Bcl9L, MLH3, WDR52, N4BP2, NKAP, PTPRF, APOA4, CP, CLU and APOE. ELISA kit was from LSBio (BIRC6, DCD, SPTBN4, HLA-DQB1, FBLN1, ANT3, VIL1), from Abnova (SERPINC1), and from USCN, China (IGK, ADCY2, Bcl9L, MLH3, WDR52, N4BP2, NKAP, PTPRF, APOA4, CP, CLU, APOE). Analyses were performed in triplicates, including positive and negative controls. A 7-point standard curve was fitted by linear regression to the O.D. values of each reference standard after subtraction of the blank using Curve Expert software version. 1.30). The concentration of the tested analyte was calculated using the regression equation and multiplied with the dilution factor.

### Proteomic secretome analysis of cervical cancer cell lines

#### Tissue culture and preparation of protein samples 

Cervical cancer cell lines, C-33A (HPV 18 positive cervical carcinoma cell lines), SiHa (HPV 16 positive cervical carcinoma cell lines), Caski (cervical carcinoma cell line derived from an intestinal metastasis) were grown to a density of 60–70 % in DMEM medium with 10 % FBS. Cells were switched to serum-free medium for 24 h at 37 °C and then washed with serum-free medium 4 times [18]. The supernatant was stored at -80 °C until further analysis. The serum-free medium supernatant was passed through a filter (pore diameter 0.22 µm) to remove dead cells and large-sized cell debris. The sample was centrifuged after the addition of a cocktail of proteinase inhibitors and then underwent ultrafiltration using a Millipore of 3 kDa. Protein amount was estimated by SDS-PAGE analysis followed by the BCA method (ThermoFisher Scientific, Waltham, MA).

#### Trypsin digestion by filter aided sample preparation (FASP) 

100 µg protein sample was brought to 100 mM DTT and 200 µL UA buffer (8 M urea, 150 mM Tris-HCl, pH 8.0) were added. The sample was transferred to a Microcon YM-30 filter unit and centrifuged at 14,000*g* for 15 min. The supernatant was discarded and 200 µL UA buffer were added, followed by centrifugation for 15 min at 14,000*g*. The following steps were each repeated twice: (1) 100 µL of 50 mM iodoacetamide (IAA) in UA was added and the sample was centrifuged for 10 min at 14,000g; (2) 100 µL of 50 mM NH_4_HCO_3_ buffer was added and the sample was centrifuged for 10 min at 14,000*g*. Finally, 40 µL trypsin buffer (2 µg trypsin in 40 µL 50 mM NH_4_HCO_3_) was added and the mixture was shaken for 1 min at 600 rpm and incubated for 16–18 h at 37 °C. The digested sample was transferred to a new collection tube. After centrifugation for 10 min at 14,000*g*, the filtrate was collected, desalted with C18-SD Extraction Disk Cartridge (3 M, United States) and quantified by measuring extinction at 280 nm in a UV spectrophotometer.

#### Liquid chromatography and tandem mass spectrometry (LC-MS/MS) 

3 replicates of 3 µg of each enzymatic digest were analyzed by LC-MS/MS using nano-LC (EASY-nLC1000 Liquid Chromatograph, ThermoFisher Scientific). Solvents A and B were 0.1 % formic acid with 2 % (A) and 84 % (B) acetonitrile, respectively. The chromatographic column (ThermoFisher Scientific EASY Column SC200 150 µm × 100 mm, RP-C18) was equilibrated with 100 % solvent A and test samples were loaded through an auto sampler at a flow rate of 300 nL/min. Peptides were eluted with linear gradients of solvent B according to the following scheme: 0–45 % (0-100 min), 45–100 % (100–108 min) and 100 % (108–120 min). Mass spectrometry was performed on a Q-Exactive (ThermoFisher Scientific) with the following settings: Positive ion mode, precursor ion scanning range: 300–1800 m/z., top 20 precursor ion selection for fragmentation by Higher Collision Energy Dissociation (HCD). MS1 resolution was 70,000 (at 200 m/z) and MS2 resolution was 17,500 (at 200 m/z).

#### Label-free analysis with MaxQuant and bioinformatic analysis of Perseus 

LC-MS/MS raw data files were imported into MaxQuant (version 1.3.0.5) for label-free quantification. The database (uniprot human_151619_20160229.fasta) contained 151,619 sequences and was downloaded on February 29, 2016. Search settings were: Main search ppm: 6; Missed cleavages: 2; MS/MS tolerance ppm: 20; De-Isotopic: TRUE; Enzyme: trypsin; Database uniprot_human_151619_20160229 fasta; Fixed modification: carbamidomethyl (cysteine); Variable modifications: Oxidation (methionine), Acetyl (Protein N-term); Decoy database pattern: reverse; LFQ: TRUE; LFQ min; ratio count: 1: Match between runs: 2 min: Peptide FDR: 0.01; Protein FDR: 0.01. The result files of MaxQuant were loaded into Perseus and processed according to the workflow (Tyanova et al. 2018). Perseus v 1.3.0.4 was used for quality control and statistical processing. All potential contaminants, reverse hits and hits only identified by site were filtered out prior to log2 transformation of the LFQ intensities. Proteins with *P* values < 0.05 were identified to be statistically significant [19, 20]. The Volcano plots were generated by MaxQuant, and *P*-values were generated by fold change and significance A/B [21].

### Validation of candidate makers by liquid chromatography and parallel reaction monitoring mass spectrometry (LC-PRM/MS)

#### Plasma sample collection 

62 samples were collected from the three groups of patients described above, including 20 samples from the normal control group, 16 samples from the HSIL or CIN II/III groups and 26 samples from early stage cervical squamous carcinoma groups, the patients’ information is shown in Additional file 1: Table S1. The collection method has been previously described [22, 23].

**Table 1:** Differences in the levels of candidate marker proteins among the patient groups

Name of protein	Peptide sequence	HSIL vs normal control			CSCC vs normal control			CSCC vs HSIL			Comparisons between the 3 groups
		Peptide ratio	*P*-value	Protein expression rate	Peptide ratio	*P*-value	Protein expression rate	Peptide ratio	*P*-value	Protein expression rate	*P*- value
sp|P23142|FBLN1-1_HUMAN	SQETGDLDVGGLQETDK	0.739	0.055	0.705	0.862	0.192	0.815	1.166	0.285	1.157	0.109
FBLN1-2_HUMAN	IIEVEEEQEDPYLNDR	0.670	0.010*		0.769	0.025*		1.148	0.364		0.015*
sp|P02649|APOE-1_HUMAN	SELEEQLTPVAEETR	1.057	0.854	0.9374	0.631	0.091	0.8355	0.597	0.053	0.9344	0.140
APOE-2_HUMAN	LAVYQAGAR	0.818	0.153		1.041	0.800		1.272	0.239		0.379
sp|P06727|APOA4-1_HUMAN	LGEVNTYAGDLQK	0.740	0.069	0.733	0.727	0.027*	0.816	0.983	0.906	1.114	0.041*
APOA4-2_HUMAN	IDQNVEELK	0.727	0.069		0.904	0.408		1.244	0.130		0.122
tr|Q6P5S8|IGKα_HUMAN	VDNALQSGNSQESVTEQDSK	0.852	0.228	0.852	0.863	0.108	0.863	1.013	0.910	1.013	0.255
Sp|P01008|ANT3-1_HUMAN	TSDQIHFFFAK	0.676	0.034*	0.622	0.808	0.096	0.759	1.196	0.273	1.222	0.052
ANT3-2_HUMAN	VAEGTQVLELPFK	0.569	0.036*		0.710	0.044*		1.249	0.232		0.025*
sp|P02647|APOA1-1_HUMAN	DLATVYVDVLK	0.963	0.886	0.851	1.081	0.744	0.959	1.123	0.661	1.127	0.890
APOA1-2_HUMAN	VSFLSALEEYTK	0.740	0.076		0.837	0.185		1.132	0.392		0.140

Additional file 6: Fig. S1 ROC curves obtained of four candidate marker proteins to distinguish between HSIL (CIN II + III) and cervical cancer (CC) and the control group (normal control, NC)

Additional file 8: Fig. S2 The concentration of four candidate marker proteins in 284 specimens from the three patient groups (CIN II + III, CC and NC)

Additional file 6: Fig. S3. Volcano Plots of (A) C33/Siha cells, (B) Siha/Caski cells and (C) C33/Caski cells

Additional file 11: Fig. S4. Numbers and percentages of 729 proteins common to the secretomes of cervical carcinoma cell lines occurring in the indicated Gene Ontology categories

#### Plasma sample preparation and enzyme digestion 

Samples were made to 10 mM DTT final concentration, IAA was added at a final concentration of 50 mM, and samples were incubated for 40 min at room temperature in the dark, followed by the addition of 100 mM ABC and LysC (100:1) and incubation for 4 h at 37 °C. Trypsin (50:1) was added followed by another incubation for 16–18 h at 37 °C. The reaction was stopped by adding an appropriate amount of 0.5 % trifluoroacetic acid (TFA). After hydrolysis, peptides were desalted using a C18 cartridge and dried, followed by resuspension in 0.1 % formic acid (FA). The peptide concentration was determined by measuring the extinction at 280 nm in a UV spectrophotometer.

#### Nano liquid chromatography (nLC) 

Equal amounts of digested peptides from each sample were mixed with equal amounts of isotope-labeled standard peptide (DSPSAPVNVTVR, underlined V represents the site of heavy isotope labeling). nLC separation was performed on an Easy nLC 1200 (ThermoFisher Scientific). Chromatographic columns were equilibrated with 95 % solvent A (0.1 % formic acid-water). After loading samples onto the trapping column, gradient separation was performed at a flow rate of 250 nL/min. Peptides were eluted with a linear gradient of solvent B (0.1 % formic acid in 84 % acetonitrile) according to the following scheme: 5–35 % (0–45 min), 35–100 (45–48 min) and 100 % (48–60 min).

#### Parallelreaction monitoring (PRM) 

PRM was performed on a Q-Exactive Plus instrument (ThermoFisher Scientific). The parameters of the first-level full MS scan were as follows: (1) Duration of analysis: 60 min; (2) Detection mode: positive ion; (3) First-level mass spectra scanning range: 400-1,100 m/z; (4) Mass spectrometer resolution: 70,000 (at m/z = 200; 5) AGC target: 3e6; 6) Maximum IT: 250 ms. After completion of the first-level full MS scan, a total of 20 PRM MS2 scans were carried out using the following parameters: (1) Isolation window: 2 Th; (2) Mass spectrometer resolution: 35,000 (at m/z = 200); (3) AGC target: 3e6; (4) Maximum IT: 200 ms; (5) MS2 activation type: HCD; (6) normalized collision energy; 27. LC-PRM/MS analysis was performed for each of the 62 samples once and Skyline (version 3.5.0) was used for the analysis of the PRM raw files.

The relative peptide ratios in the cancer vs. control samples were calculated by dividing the mean of the peptide area in the cancer samples by the mean of the peptide area in the normal control samples. A Student’s *t*-test was used to estimate the significance of the peptide changes observed. Parallel reaction monitoring data were also processed with Skyline software after selecting transitions with dotp values matching the library spectrum with a value of ≥ 0.9.

### Total RNA isolation and quantitative real‐time PCR (qPCR)

Total RNA was isolated using TRIzol reagent (Invitrogen,USA), and cDNA was generated using the Hifair®II1st Strand cDNA Synthesis SuperMix (Yeason, China). qPCR was performed using a 7500 system (ABI, USA) with Hieff qPCR SYBR Green Master Mix (Yeason, China). The relative expression levels of the target genes were normalized to the expression level of GAPDH. The data analyses were performed using the 2^−ΔΔCt^ method. The primer sequences are provided in Additional file 2: Table S2.

### Western blotting

Cultured cells were lysed with lysis buffer. Equal amounts of protein were run on 10 % SDS-PAGE and electrotransferred to polyvinylidene fluoride membranes (Millipore). After blocking with 5 % milk in TBST, membranes were incubated with primary antibodies overnight. The following antibodies were used: anti-ANT3 (1:1000, Proteintech, 14B41-1-AP), anti- FBLN1 (1:1000, Bioss, bs-0809R), E6/E7 (Abcam; ab70, ab30731) and anti-GAPDH (1:6000, Yeasen). Membranes were then incubated with the Rabbit peroxidase-conjugated secondary antibody (1:10,000, Abclonal). The blots were detected by sensitive chemiluminescence liquid (Yeasen), Biorad software was used to captured the images.

### E6/E7 knockout by CRISPR/Cas9 gene editing

CRISPR/Cas9 single guide sequences specifically targeting HPV16 infection marker E6/E7 (sg E6/E7) were designed from Vector Builder (www.vectorbuilder.com) and produced from Cyagen Company. The E6/E7 gRNA guide sequence used 5'-CAACAGTTACTGCGAC GTG-3' and 5'-TCCGGTTCTGCTTGTCCAGC-3'. Siha cell lines were transduced twice with firstly sgRNAE6/E7 lentiviruses, EGFP lentiviruses are as positive control and transfection reagent as negative control. Puromycin (Merck, German) was added on day 4 by using the minimal toxic dose to select transduced cells. Then after 3 days of screening and observing the well condition of cells, the second transduction was performed with Cas9 Lentiviruses. At this time, hygromycin in minimal toxic dose was added after 3 days. The second screening finished after all negative control cells were died. To minimize off-target situation, monoclonal screening was conducted, Single colonies were spread in 96-well plate and selected by colony formation assay. The effect of E6/E7 gene editing on cell proliferation was assessed by Trypan Blue viable cell counting over a 7-day time course. The efficiency of E6/E7 knockout was proved by qPCR and western blotting.

### Immunofluorescence

For immunofluorescence staining, Confocal dish (Nest, China) with planted cells were washed thrice with PBS and then incubated with 4 % Polyformaldehyde to fixation for 15 min. Then washed with PBS and treated with PBS containing 0.25 % Triton-X (Solabio, China) for20 min, blocked with 5 % Goat serum (Solabio, China) for 30 min in 37℃, and incubated with primary antibody (anti-ANT3, Proteintech, 14B41-1-AP), (anti- FBLN1, Bioss, bs-0809R) diluted in PBS overnight. Next, followed by washed thrice and incubated with proper secondary antibodies. Finally, the slides were incubated with DAPI, dehydrated, and mounted them for further evaluation in microscope.

### Statistical analysis

All experiments were repeated at least three times. In the PRM-MS experiment, two different peptide markers were designed for each protein for quantitative detection. Statistical evaluation was conducted with Graphpad Prism (version 7.00) and IBM SPSS statistical software (version 24.0). The Shapiro-Wilk W test was used to determine whether the data were normally distributed continuous variables. Levene’s test was used to justify equality of variances. A *t*-test was used to analyze mean values for normally distributed continuous variables, and the Mann-Whitney U test was employed to compare mean values for non-normally distributed continuous variables. For all statistical tests, *P* < 0.05 (two-tailed test) was considered statistically significant.

## Results

### HPV status and subtypes in the cervical cancer patient cohort

Cervical exfoliated cells and matching serum samples were collected from a total of 284 patients with various cervical lesions from 2013 to 2016 in the Xinjiang province of China. Additional file 3: Table S3  shows the clinical information and proportion of individual cervical lesions, among which 75 (26.41 %), 88 (30.99 %) and 121 (42.61 %) were from patients with normal control, precancerous lesions (HISL or CIN II/III) and cervical squamous carcinoma at an early stage (< IIa). HPV positivity was lower in patients with normal control than in patients with precancerous lesions and cervical squamous cancer. The cohorts also differed with respect to the frequency of high-risk HPV subtypes (Additional file 3: Table S3). High risk subtypes, including HPV 16, and 58 were considerably more frequently detected in patients with precancerous lesions and early-stage cervical squamous carcinoma than in patients with normal control, whereas HPV 18, 33, 52, 31 and 53 were more frequently detected in early-stage cervical squamous carcinoma than in precancerous lesions and HPV 51 and 58 solely occurred in early-stage cervical squamous carcinoma. This result suggested that normal control could be differentiated from precancerous lesions and early-stage cervical squamous carcinoma by the different incidence of HPV high-risk subtypes 16 and 18. We therefore quantitatively measured subtype specific HPV antibody serum levels by ELISA.

### Quantitative measurement of HPV antibody serum levels

HPV antibody serum levels were determined with the HR-HPVL1 (IgG) ELISA method. As shown in Additional file 4: Table S4, HPV-16 L1 antibody was detected in 50.4 % (61/121), 43.2 % (38/88) and 42.7 % (32/75) of patients with early-stage cervical carcinoma, precancerous lesions (CIN II/III) and normal control reference. However, these differences showed a high p value (0.46), indicating weak evidence against the null hypothesis that antibodies against high risk papilloma virus L1-capsid cannot differentiate between various cervical lesions. Thus, the serum levels of HPV-L1 antibody are not significantly related to the progression of cervical cancer.

### Validation of four candidate marker proteins in serum

Expression of several candidate marker proteins was measured in specimens from the cohort of 284 patients, including CLU, ADCY2, APOA4, APOE, BCI9L, CP, IGK, N4BP2, PTPRF, WDR52, MLH3, NKAP and SERPIN. Significant differences between the three patient groups were found for four proteins: CLU, APOA4, APOE and MLH3 (*P* < 0.05) (Additional file 5: Table S5). There was no significant difference in the expression of the other proteins.

Next, the co-occurrence of these four proteins with HPV infection was determined. The co-occurrence rate of HPV with APOA4, MLH3, CLU and APOE was 54.9 % (156/284), 71.1 % (202/284), 62.7 % (178/284) and 51.1 % (145/284), respectively (Additional file 6: Fig. S1).

We then determined the diagnostic ability of APOA4, MLH3, CLU and APOE to classify cervical cancer, CIN II + III and the normal control group by calculating receiver operating characteristics (ROC) curves, which showed that only APOE can detect CIN II + III (85.7 % sensitivity and 53.9 % specificity). For cervical carcinoma, MLH3 had some diagnostic ability, but its sensitivity was poor (35.9 %) despite high specificity (96.15 %). (Additional file 7: Table S6 ).

Overall, none of the four markers showed a profile that would allow clinical application due to insufficient sensitivity and specificity and large variability (Additional file 8:  Fig. S2).

### Secretome analysis of three cervical carcinoma cell lines by label‐free quantitative proteomics

**Fig. 2:**
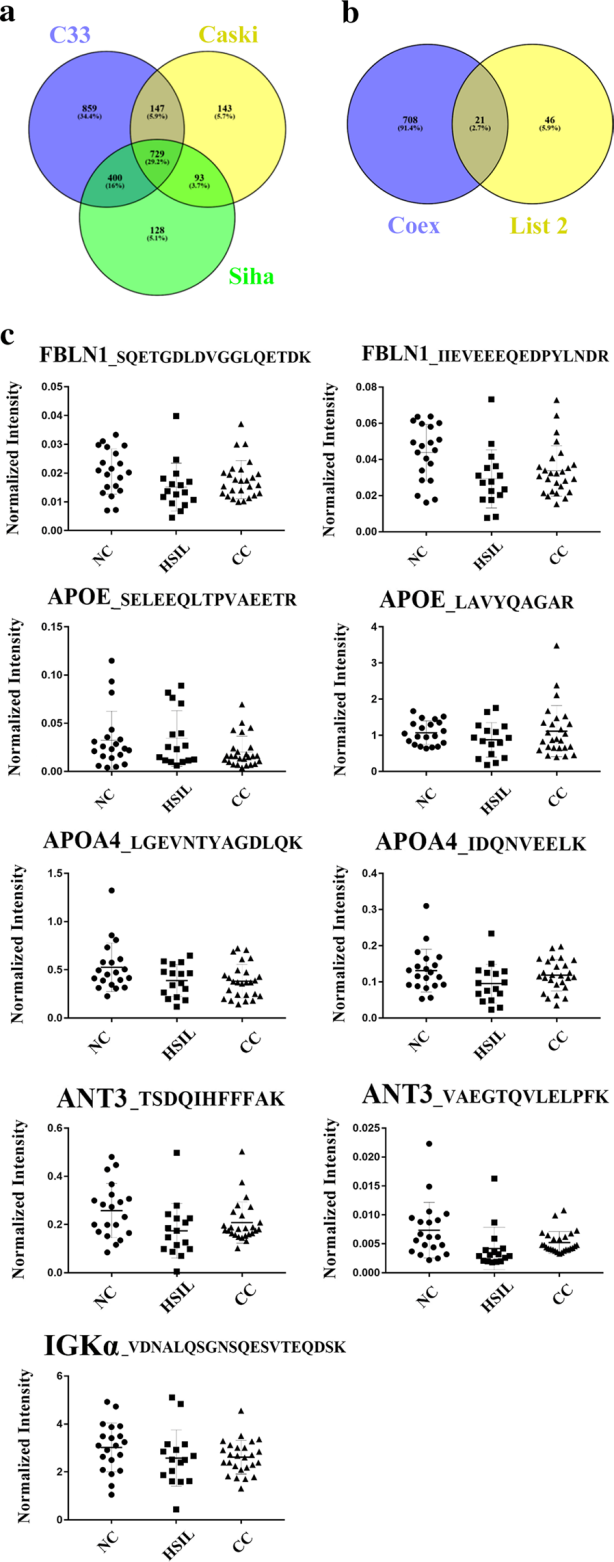
Overview of the secretome analysis. **a** The number of protein identified in each cell line and the overlap in the datasets are indicated in a Venn diagram.** b** Overlap between the shared secretome of the cervical carcinoma cell lines (729 proteins) and a list of 46 proteins previously identified by proteomic analysis of cervical cancer tissues (Additional file 9: Table S7). **c** Levels of FBLN1, APOE, APOA4, ANT3 and IGKα in the three ptient groups as determined by LC-PRM/MS

Reasoning that secretory proteins may constitute a potential source of novel candidate disease marker proteins, we employed label-free quantitative proteomics to profile secretory proteins from C-33A, Caski, and SiHa cervical carcinoma cell lines. As shown in Fig. 2a, Additional file 9: Table S7 and  Additional file 10: Fig. S3, a total of 729 proteins were common to the secretomes of all three cell lines. These proteins fell into 47 different Gene Ontology categories according to the biological processes, molecular functions and cellular components they are involved with (Additional file 11: Fig. S4). As expected for secreted proteins, almost 80 % of the proteins were associated with the GO term “extracellular region”. There was an overlap of 21 proteins between the shared secretome of the cervical carcinoma cell lines (729 proteins) and a list of 46 proteins previously identified by proteomic analysis of cervical cancer tissues (Additional file 9: Table S7; Fig. 2b). Finally, six candidate proteins (FBLN1, APOE, APOA4, GRP75, ANT3 and APOA1) were identified after combining the secretome analysis with the ELISA data. These proteins were further validated as potential prognostic indicators of HPV infection.

### Validation of candidate marker proteins using LC-PRM/MS

**Fig. 3:**
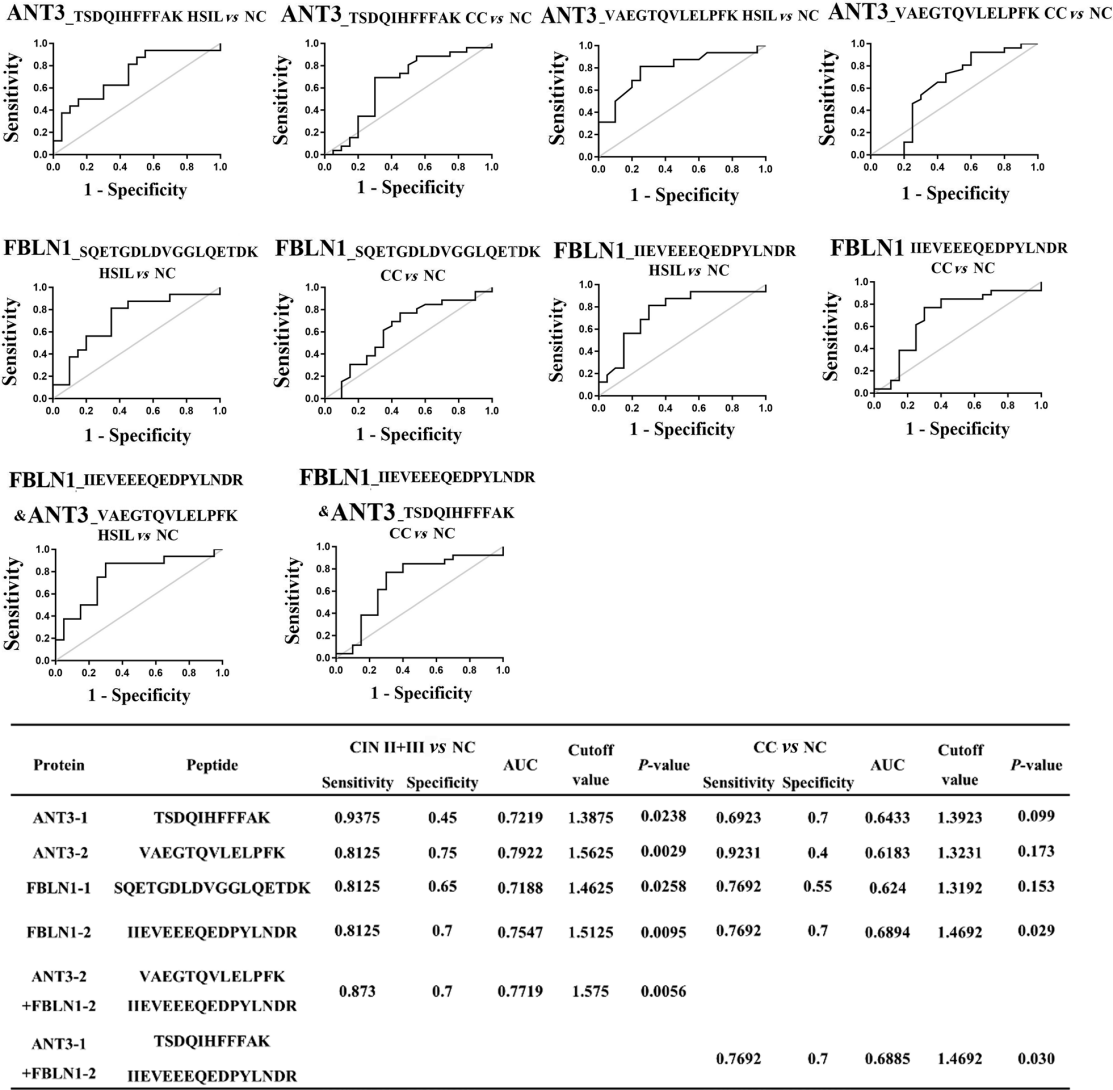
ROC curves showing the sensitivity and specificity, the area under the curve, and the combined effect to assess the diagnostic abilities of peptides and peptide combinations derived from ANT3 and FBLN1 proteins to distinguish between HSIL vs. NC and CC vs. NC

As shown in Additional file 13: Table S8 and Fig. 2 C, proteotypic peptides from each of the six candidate proteins from 12 specimens were analyzed by LC-PRM/MS to quantitatively measure the serum levels of these proteins in the three patient cohorts. An isotopically labeled peptide (DSPSAPVNVTVR) was included as an internal standard. FBLN1 and ANT3 levels were increased in the precancerous lesions (HSIL) and in the CC groups (*P* < 0.05, Table 1). CC exhibited increased levels of APOA4 compared with the normal control group (NC) (*P* < 0.05). Although both the HSIL and CC appeared to have increased expression of APOE and APOA1 compared with NC, the differences had *P* values > 0.05 (Fig. 3).

### Validation of the expression of ANT3 and FBLN1in Vitro and the relationship with HPV

Next, the co-occurrence of differences in serum protein levels and HPV infection was analyzed. FBLN1, ANT3, APOA4 and APOE showed co-occurrences of 75.7 % (215/284), 85 % (241/284), 67.9 % (193/284), 51.1 % (145/284), respectively.

**Fig. 4:**
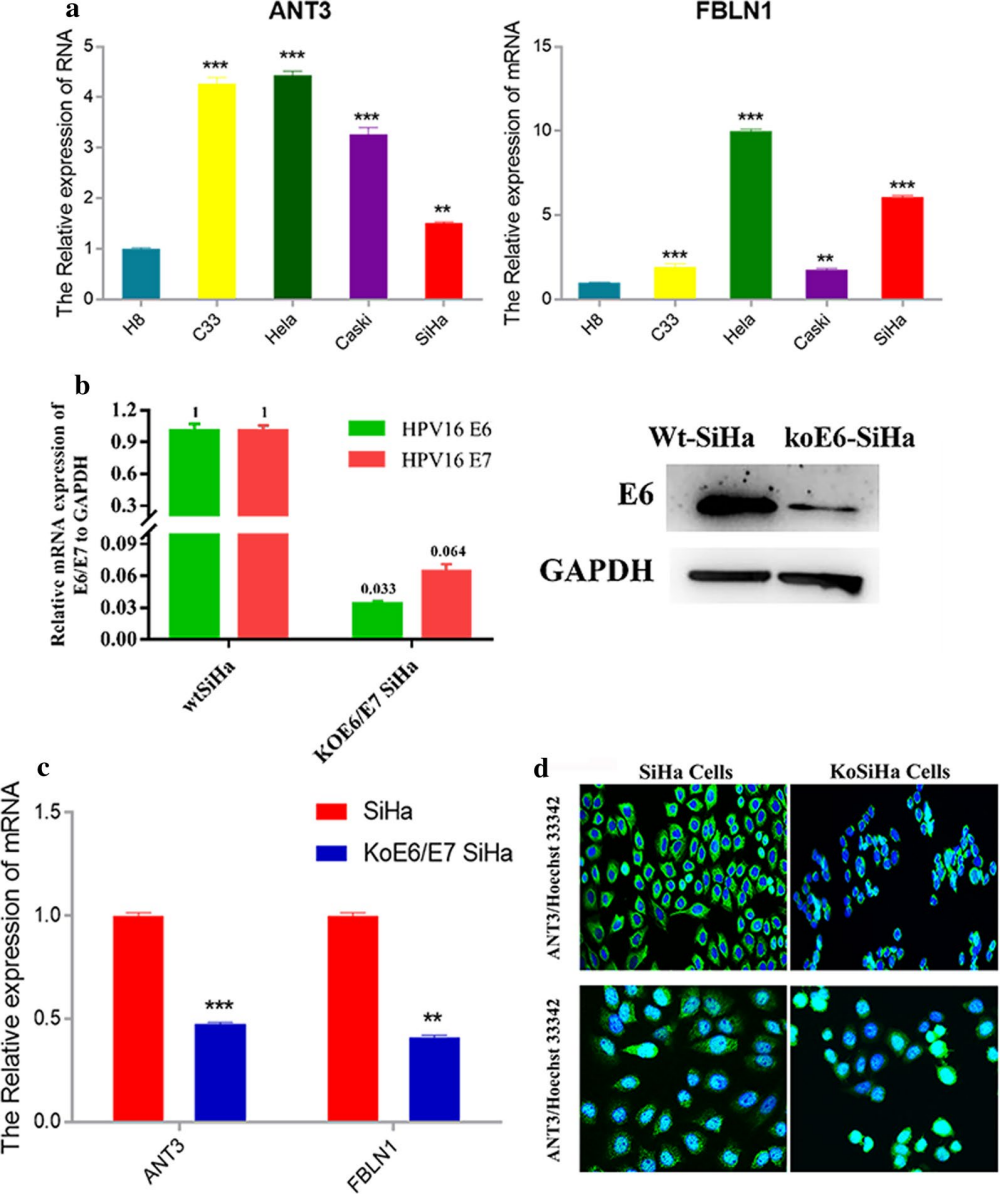
**a** The relative mRNA expressions of ANT3 (Left) and FBLN1 (Right) among cervical cancer cell lines- H8, C-33A, Hela, Caski and SiHa. **b** (Left) Transcript levels between wide-type SiHa cells and KoE6/E7 SiHa cells for HPV16E6 and HPV16E7. (Right) Western blotting in wide-type SiHa cells and KoE6/E7 SiHa cells for expression of HPV16E6. ** C** ANT3 and FBLN1 transcript levels in wide-type SiHa cells and KoE6/E7 SiHa cells respectively. Data are mean ± s.e.m. * *P* < 0.05, * * *P* < 0.01, * * * *P* < 0.001, two-tailed t-test. ** d**. Representative images of the expressions of ANT3 (Upper panel) and FBLN1 (Lower panel) between wide-type SiHa cells and KoE6/E7 SiHa cells

The nature expression of ANT3, FBLN1 in vitro were investigated by qPCR and western blotting in the wide type cervical cancer cell lines, SiHa (HPV16^+^), Hela (HPV18+), Caski (HPV16^+^, HPV18^+^), C-33A (HPV^−^) as well as H8 (Immortalized ceivical epithelial cells). We observed that HPV-positive cervical cell lines (HeLa/SiHa/Caski) had a higher content with ANT3 and FBLN1 compared with H8 cells and C-33A (HPV^−^) (Fig. 4a).

After that, we used CRISPR/Cas9 gene editing lentivirus to transfect cell line Siha to knock out E6/E7, which symbolized the infection of HPV16. The efficiency of knock out E6/E7 by qPCR was 96.7 % and 93.6 %. Furthermore, the result of western blotting showed that the expression of E6 protein was knockdown (Fig. 4b). However after the loss of E6/E7, the content of ANT3 and FBLN1 in KoE6/E7 SiHa were downregulated compared to wide type SiHa by qPCR and immunofluorescence, which indicated the expression of ANT3 and FBLN1 in cervical cancer may be affected by HPV infection (Fig. 4 c and Fig. 4d).

## Discussion

Biomarkers can provide rich clinical information for early diagnosis as well as therapeutic and follow-up strategies. Therefore, screening of serum protein markers that are associated with HPV infection is expected to enable early detection of cervical carcinoma and prediction of tumor incidence in patients with persistent HPV infection. The search for and development of suitable biomarkers is generally divided into three phases, namely their identification, verification and clinical validation [24].

In the present study, we applied ELISA, secretome analysis from various cervical carcinomas, and PRM-MS for relative and absolute quantitative validation of previously identified candidate protein markers in patient serum. We identified two proteins (FBLN1 and ANT3), which were closely related to cervical precancerous lesions and cervical carcinoma. Moreover, these proteins were associated with HPV infection and HPV antibody levels [25]. Our study suggests that FBLN1 and ANT3 can be used as general serum protein markers for cervical carcinoma and HPV infection thus warranting the future development of ELISA kits for the detection of FBLN1 (IIEVEEEQEDPYLNDR) and ANT3 (VAEGTQVLELPFK) peptides.

Serum secretory proteomics, namely secretomics, can detect secreted or leaking proteins, which originate from various tissues and cells and thus reflect multiple biological processes in the body. With the rapid development of serum secretory proteomics in the oncology field, a number of new serum protein markers have been identified that are associated with tumor development and progression [26]. Chang et al. [27] examined secretory proteins in the nasopharyngeal carcinoma cell line, TW 04, and found significantly increased expression of CLIC1, which is a chloride channel protein. Wu et al. [28] identified collapsin response mediator protein 2 (CRMP2) in 21 colon cancer cell lines of various origins when screening and optimizing for colon cancer specific secretory proteins. Detection of CRMP2 in patient serum using ELISA led to a diagnostic sensitivity of 60.5 %, which was greater than that of carcino-embryonic antigen (CEA). In the present study, various cervical carcinoma cell lines with different characteristics, including HeLa (HPV 18 + cervical carcinoma cell line), SiHa (HPV 16 + cervical carcinoma cell line) and Caski (intestinal metastatic cervical carcinoma cell line), were cultured in serum-free medium and their secretory proteins were profiled for the identification of candidate protein markers. The tumor specificity of these proteins was further verified and those closely associated with cervical carcinoma were screened as candidate serum protein biomarkers.

Although it is known that cervical carcinoma and precancerous lesions are closely related with HPV infection, HPV-related proteomics is still in its infancy [29]. The detection rate of high-risk HPV subtypes (such as HPV 16 or 18) in Uygur women with cervical carcinoma is high (78 ~ 88 %), approaching the level of cervical carcinoma in high incidence areas [30]. Previous studies have demonstrated that carcinogenic proteins such as E5, E6 and E7, encoded by a high-risk subtypes probably affect host regulation of gene expression and various biological processes, such as DNA damage repair, cell-cycle regulation and antigen presentation. As a result, infected cells might proliferate abnormally and evade the immune response, thus promoting tumor initiation [31]. The human is thought to clear most HPV infections and only persistent infection with high-risk HPV enabled by immune escape may drive cervical carcinoma [32]. Although HPV vaccination has been implemented in developed countries, the high costs limit its use in underdeveloped areas of the world. Therefore, serum tumor biomarkers for early detection should consider the severity and type of HPV infection. Additional file 4: Table S4 shows that the plasma levels of HPV-L1 antibody is not significantly related to the progression of cervical cancer, indicating that the absence of HPV-L1 antibody signifies HPV escape from immunologic surveillance and immune clearance.

In addition, FBLN1 is located on the long arm of chromosome 22 (22q13) and its product is a secreted glycoprotein involved in the formation of the fibrous extracellular matrix [33, 34]. The protein products of the FBLN gene family are extracellular matrix proteins (ECM), which are components of the basement membrane and elastic fibers, which play important roles in the maintenance of tissue structure. FBLN1 is closely related to migration, adhesion, and invasion of tumor cells. FBLN1 exists in the proteins isolated from the mammary library and interacts with the AF1 and DBD domains of the estrogen receptor β (ERβ). Adenine nucleotide translocases (ANTs) are abundant proteins in the inner mitochondrial membrane which catalyzes the exchange of cytoplasmic ADP with mitochondrial ATP. In the current study, ELISA and PRM-MS were applied to demonstrate that the expression of FBLN1 and ANT3 increase in cervical carcinoma and precancerous lesion groups, in close relationship with HPV status and the carcinogenic protein E6/E7, showing co-occurrences of 75.7 % and 85 %, respectively. These results strongly suggest that FBLN1 and ANT3 may be used as protein biomarkers for both cervical carcinoma and HPV infection with higher sensitivity and specificity by using the peptide of FBLN1 (IIEVEEEQEDPYLNDR) or ANT3 (VAEGTQVLELPFK) Additional file 13: Figure S5.

## Conclusions

We verified FBLN1 and ANT3 as promising early indicators of HPV-associated cervical carcinoma. The findings are expected to have considerable impact on investigating the progression of cervical lesions, revealing underlying pathogenic mechanism(s), and for prophylaxis of cervical carcinoma.

## Supplementary Information

**Additional file 1: Table S1.**

**Additional file 2: Table S2.** List of ANT3 and FBLN1 primer sequences for qPCR.

**Additional file 3: Table S3. **HPV subtype frequencies in the patient cohorts.

**Additional file 4: Table S4.**Distribution of cases with HPV 16 L1 serum antibodies.

**Additional file 5: Table S5. **Levels of candidate marker proteins in the three patient groups.

**Additional file 6: Figure S1**.

**Additional file 7: Table S6.**Preliminary screening of CLU, APOA4, APOE and MLH3 using ELISA.

**Additional file 8: Figure S2.**Preliminary screening of CLU, APOA4, APOE and MLH3 using ELISA.

**Additional file 9: Table S7**.

**Additional file 10: Figure S3.**.

**Additional file 11: Figure S4.**.

**Additional file 12: Table S8.**List of target protein and peptide sequences for PRM quantification.

**Additional file 12: Figure S5.**.

## Data Availability

The datasets generated and/or analysed during the current study are available in the [ProteomeXchange via the PRIDE] repository, [http://www.coxdocs.org/doku.php?id=perseus:user:use_cases:interactions].

## Abbreviations

2D-DIGE: 2-dimensional difference gel electrophoresis
ANTs: Adenine nucleotide translocases
CC: Cervical carcinoma
CEA: Carcino-embryonic antigen
CESC: Cervical squamous carcinoma
CIN: Cervical intraepithelial neoplasia
CRMP2: Collapsin response mediator protein 2
ECM: Extracellular matrix proteins
ELISA: Enzyme-linked immunosorbent assay
Erβ: Estrogen receptor β
FA: Formic acid
FASP: Filter aided sample preparation
HCD: Higher Collision Energy Dissociation
HR-HPVL1: High risk Papilloma Virus L1-Capsids
HSIL: High-grade squamous intraepithelial lesion
IAA: Iodoacetamide
LC-MS/MS: Liquid chromatography and tandem mass spectrometry
LC-PRM/MS: Liquid chromatography and parallel reaction monitoring mass spectrometry
MALDI-TOF-MS: Matrix-Assisted Laser Desorption/Ionization Time of Flight Mass Spectrometry
nLC: Nano liquid chromatography
PRM-MS: Parallel reaction monitoring mass spectrometry
qPCR: Quantitative real-time PCR
ROC: Receiver operating characteristics
TCGA: The Cancer Genome Atlas
TFA: Trifluoroacetic acid
