# Supplementary material for: Discovery and validation of FBLN1 and ANT3 as potential biomarkers for early detection of cervical cancer
Source: Cancer Cell Int. 2021 Feb 18;21:125. doi: 10.1186/s12935-021-01802-5 (PMC7893763; doi:10.1186/s12935-021-01802-5)
Supplement: Supplementary file 2 — Additional file 2: Table S2. List of ANT3 and FBLN1 primer sequences for qPCR. [file 12935_2021_1802_MOESM2_ESM.docx]

**Additional file 2: Table S2. List of ANT3 and FBLN1 primer sequences for qPCR**

| **Gene name** | **Primer Sequence 5‘-3’** |
| --- | --- |
| FBLN1 For | TGCGAATGCAAGACGG |
| FBLN1 Rev | CGTAGACGTTGGCACA |
| ANT3 For | GGACTAGTCCGATTCCGTGTCTTGAT |
| ANT3 Rev | CCCAAGCTTTTGACCTCTGCGTCCTCT |
| HPV16E6 For | CAATGTTTCAGGACCCACAGG |
| HPV16E6 Rev | CTCACGTCGCAGTAACTGTTG |
| HPV16E7 For | CGGGATCCATGCATGGAGATACA |
| HPV16E7 Rev | GCG­GGC­CCT­TAT­GGT­TTC­TGA­GA |
| GAPDH For | AGCCACATCGCTCAGACAC |
| GAPDH Rev | GCCCAATACGACCAAATCC |
