# Supplementary material for: Discovery and validation of FBLN1 and ANT3 as potential biomarkers for early detection of cervical cancer
Source: Cancer Cell Int. 2021 Feb 18;21:125. doi: 10.1186/s12935-021-01802-5 (PMC7893763; doi:10.1186/s12935-021-01802-5)
Supplement: Supplementary file 3 — Additional file 3: Table S3. HPV subtype frequencies in the patient cohorts. [file 12935_2021_1802_MOESM3_ESM.docx]

**Additional file 3: Table S3. HPV subtype frequencies in the patient cohorts**

| **HPV infection** | **HPV genotype** | **Cervicitis (N, %)** | **Precancerous**  **lesions (N, %)** | **Early-stage cervical**  **squamous carcinoma (N, %)** | **Total (N, %)** |
| --- | --- | --- | --- | --- | --- |
| **HPV positive** |  | **23 (30.67)** | **69 (78.41)** | **93 (76.86)** | **185 (65.14) ^a^** |
| HPV high-risk subtype single infection |  | 16 (69.57) | 53 (76.81) | 78 (83.87) | 147 (79.46) |
| HPV high-risk subtype multiple infection |  | 7 (30.43) | 16 (23.19) | 15 (16.13) | 38 (20.54) |
|  | hrHPV subtype of 16 | 11 (14.67) | 38 (43.18) | 54 (44.63) | 104 (36.62) |
|  | hrHPV subtype of 18 |  | 9 (10.23) | 14 (11.57) | 23 (8.10) |
|  | hrHPV subtype of 58 | 6 (8%) | 8 (9.09) | 5 (4.13) | 19 (6.69) |
|  | hrHPV subtype of 33 |  | 1 (1.14) | 3 (2.48) | 4 (1.41) |
|  | hrHPV subtype of 52 |  | 1 (1.14) | 2 (1.65) | 3 (1.06) |
|  | hrHPV subtype of 31 |  | 2 (2.27) | 3 (2.48) | 5 (1.76) |
|  | hrHPV subtype of 53 |  | 2 (2.27) |  | 2 (0.70) |
|  | hrHPV subtype of 51 |  |  | 1 (0.83) | 1 (0.35) |
|  | hrHPV subtype of 68 |  |  | 1 (0.83) | 1 (0.35) |
|  | HPV subtype of 66 | 2 (2.67) | 1 (1.14) |  | 3 (1.06) |
|  | HPV subtype of 45 | 1 (1.33) |  | 1 (0.83) | 2 (0.70) |
|  | HPV subtypes of 56 | 1 (1.33) |  |  | 1 (0.35) |
| **HPV negative** |  | **52 (69.33)** | **19 (21.59)** | **18 (14.88)** | **99 (34.86) ^b^** |
| **Total** |  | **75 (26.41)** | **88 (30.99)** | **121 (42.61)** | **284 (100.00) ^c^** |

Note: hrHPV refers to high risk HPV subtypes
