# Supplementary material for: Discovery and validation of FBLN1 and ANT3 as potential biomarkers for early detection of cervical cancer
Source: Cancer Cell Int. 2021 Feb 18;21:125. doi: 10.1186/s12935-021-01802-5 (PMC7893763; doi:10.1186/s12935-021-01802-5)
Supplement: Supplementary file 4 — Additional file 4: Table S4.Distribution of cases with HPV 16 L1 serum antibodies. [file 12935_2021_1802_MOESM4_ESM.docx]

## Additional file 4: Table S4. Distribution of cases with HPV 16 L1 serum antibodies

| **Group** | **Case** | **Cases positive for HPV 16 L1 antibody** | **Positivity rate (%)** |
| --- | --- | --- | --- |
| Cervical carcinoma | 121 | 61 | 50.4 |
| HSIL | 88 | 38 | 43.2 |
| Reference group (cervicitis) | 75 | 32 | 42.7 |
| Total | 284 | 131 | 46.13 |
| *P*-value (Three-way comparison) |  | 0.4600 |  |
