# Supplementary material for: Discovery and validation of FBLN1 and ANT3 as potential biomarkers for early detection of cervical cancer
Source: Cancer Cell Int. 2021 Feb 18;21:125. doi: 10.1186/s12935-021-01802-5 (PMC7893763; doi:10.1186/s12935-021-01802-5)
Supplement: Supplementary file 5 — Additional file 5: Table S5. Levels of candidate marker proteins in the three patient groups. [file 12935_2021_1802_MOESM5_ESM.docx]

## Additional file 5: Table S5. Levels of candidate marker proteins in the three patient groups

| **Candidate** | **NC** (ng/ml)  **(75, 26.41%)** | **CIN II+III** (ng/ml)  **(88, 30.99%)** | **CC** (ng/ml)  **(121, 42.61%)** | ***P*-value** | **F-value** |
| --- | --- | --- | --- | --- | --- |
| CLU (ng/ml) | 98289.16+38303.7 | 85896.09+31957.98 | 119608.13+62983.9 | 0.016* | 4.34 |
| ADCY2 (ng/ml) | 1.71+1.47 | 2.46+5.06 | 3.43+6.25 | 0.39 | 0.97 |
| APOA4 (ng/ml) | 2.0367E7+9.1952E6 | 2.2136E7+1.0095E6 | 2.6079E7+1.9821E6 | 0.044* | 2.27 |
| APOE (ng/ml) | 66995.76+30717.14 | 92540.88+42705.64 | 83814.66+46611.11 | 0.039* | 2.38 |
| BCI9L (ng/ml) | 2.31+1.71 | 2.05+1.21 | 4.13+5.12 | 0.053* | 3.05 |
| CP (ng/ml) | 785157.86+29861.06 | 792136.92+29166.23 | 781602.49+24236.97 | 0.35 | 1.08 |
| IGKα (ng/ml) | 15211.19+5624.99 | 16154.95+5357.13 | 18242.80+1080.08 | 0.14 | 2.03 |
| N4BP2 (ng/ml) | 1.90+3.84 | 1.0388+0.65 | 1.61+2.02 | 0.50 | 0.70 |
| PTPRF (ng/ml) | 31.80+25.06 | 40.66+32.60 | 34.16+32.09 | 0.59 | 0.53 |
| WDR52 (ng/ml) | 2.52+1.59 | 2.03+0.91 | 2.00+1.29 | 0.25 | 1.40 |
| MLH3 (ng/ml) | 0.37+0.31 | 0.43+0.30 | 0.94+1.14 | 0.009* | 4.93 |
| NKAP (ng/ml) | 1.92+1.63 | 1.79+0.40 | 1.97+1.11 | 0.85 | 0.16 |
| SERPIN (ng/ml) | 366.14+99.41 | 399.39+53.93 | 367.50+87.13 | 0.31 | 1.18 |
