# Supplementary figures and images for: Discovery and validation of FBLN1 and ANT3 as potential biomarkers for early detection of cervical cancer
Source: Cancer Cell Int. 2021 Feb 18;21:125. doi: 10.1186/s12935-021-01802-5 (PMC7893763; doi:10.1186/s12935-021-01802-5)

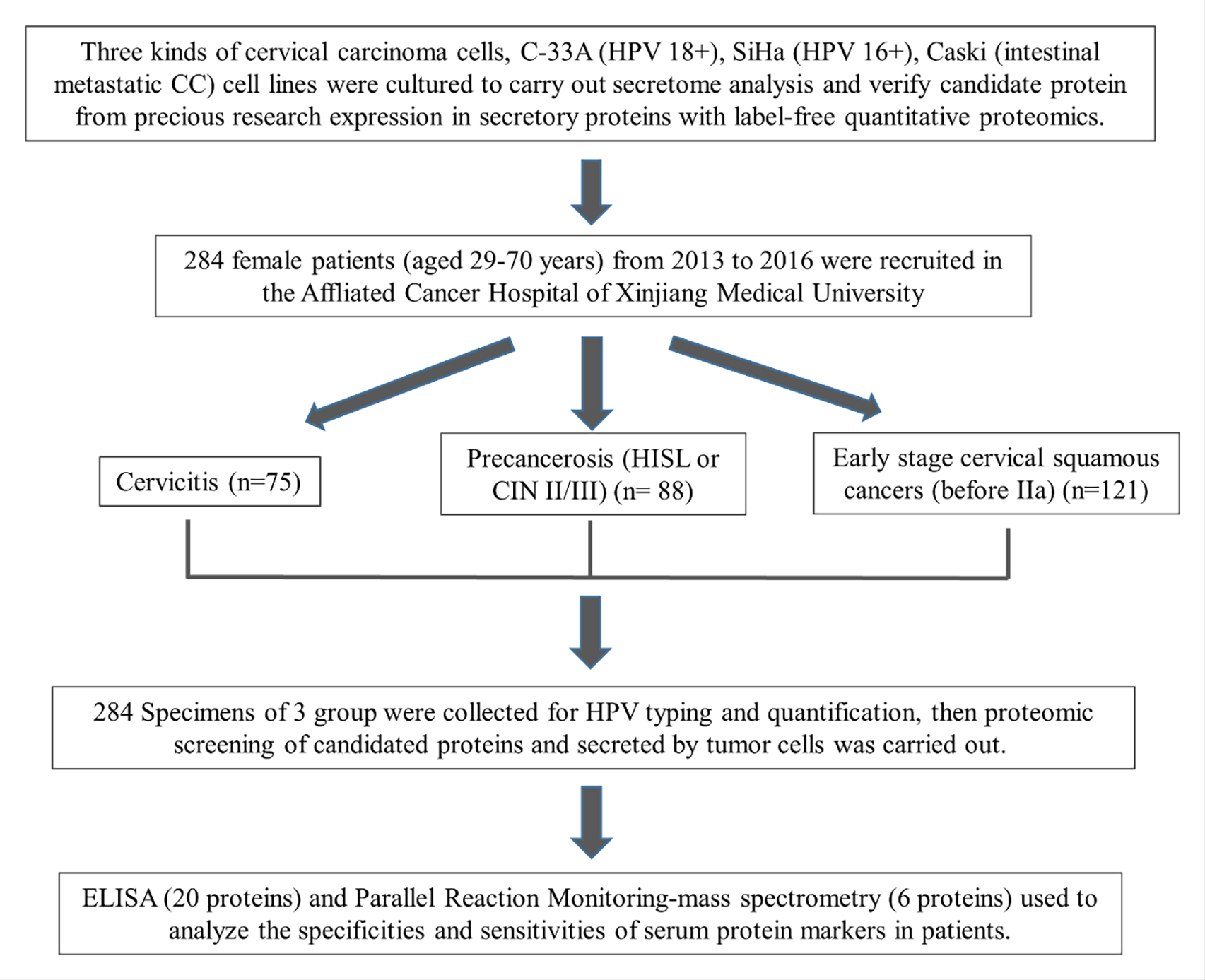

Supplement: Supplementary file 6 — Additional file 6: Figure S1. [file 12935_2021_1802_MOESM6_ESM.tif]

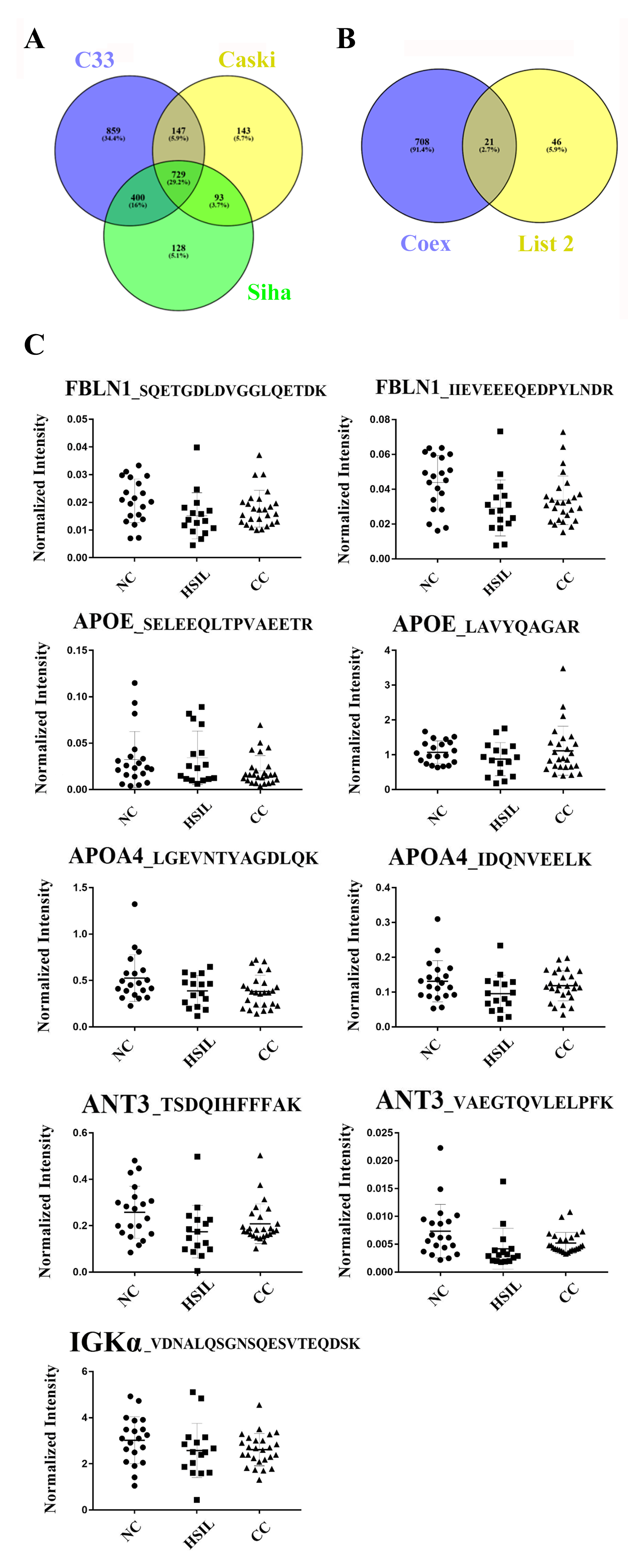

Supplement: Supplementary file 8 — Additional file 8: Figure S2.Preliminary screening of CLU, APOA4, APOE and MLH3 using ELISA. [file 12935_2021_1802_MOESM8_ESM.tif]

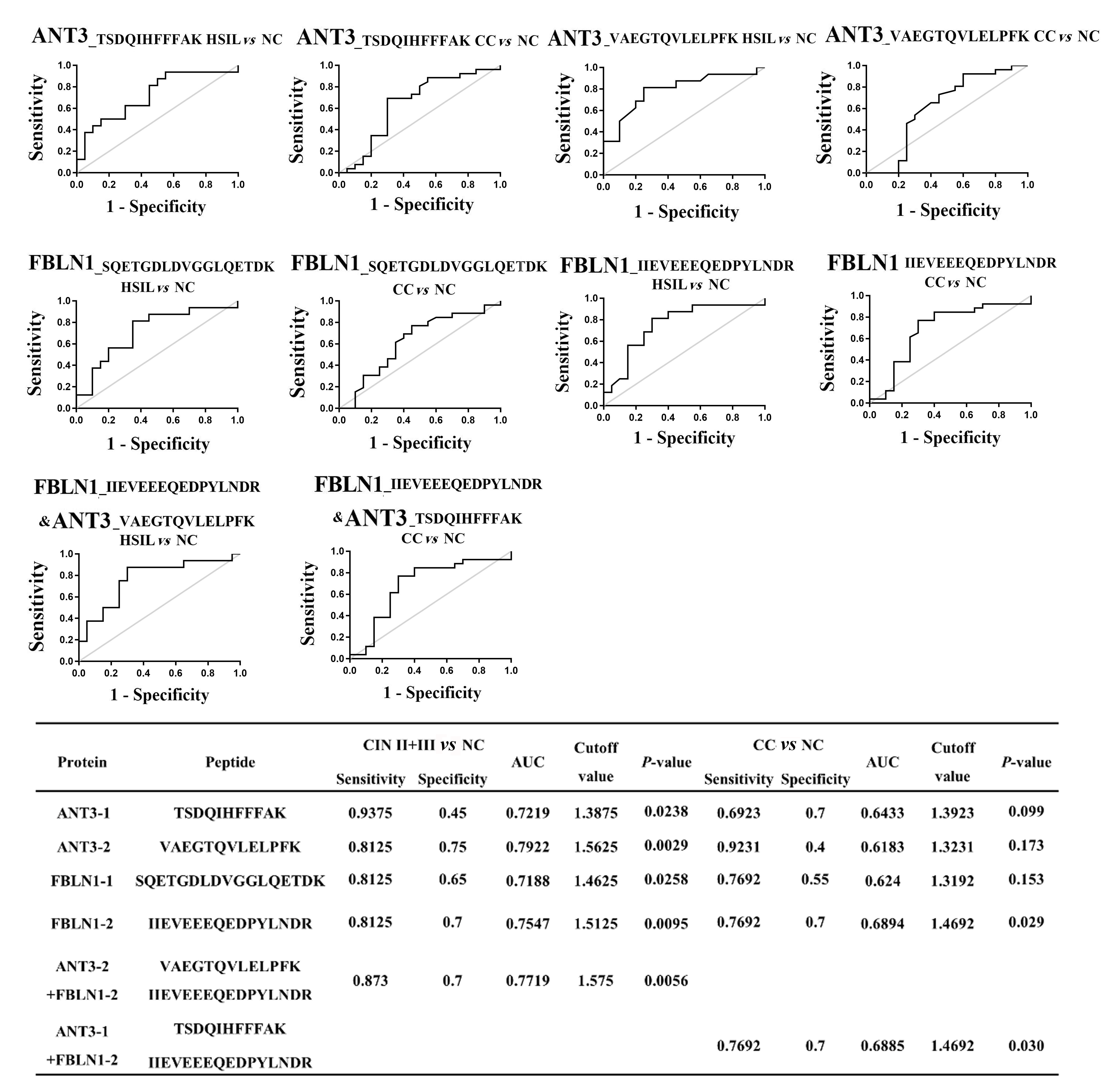

Supplement: Supplementary file 10 — Additional file 10: Figure S3.. [file 12935_2021_1802_MOESM10_ESM.tif]

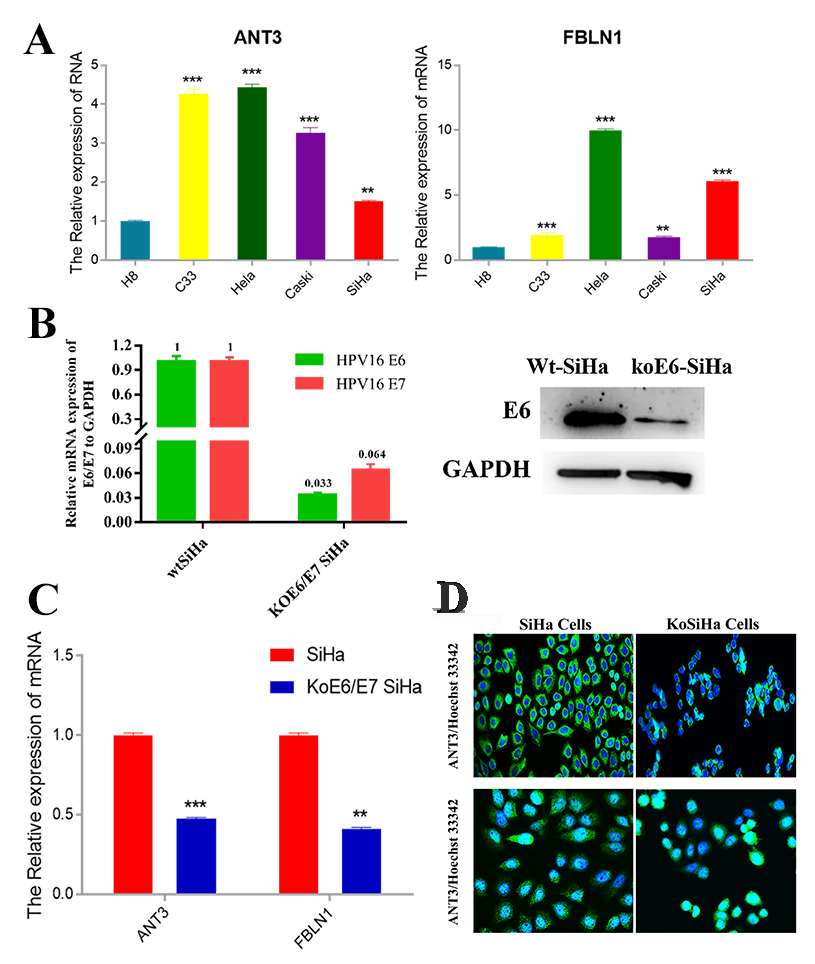

Supplement: Supplementary file 11 — Additional file 11: Figure S4.. [file 12935_2021_1802_MOESM11_ESM.tif]

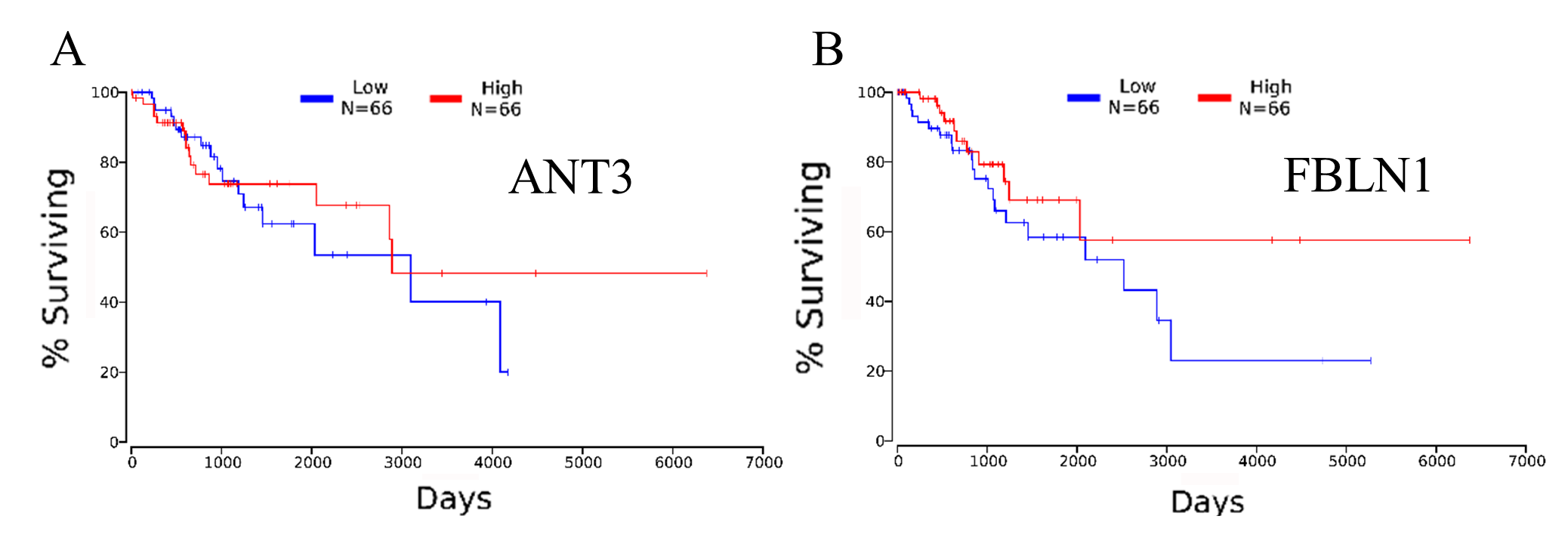

Supplement: Supplementary file 13 — Additional file 12: Figure S5.. [file 12935_2021_1802_MOESM13_ESM.tif]
