# Supplementary material for: Discovery and validation of FBLN1 and ANT3 as potential biomarkers for early detection of cervical cancer
Source: Cancer Cell Int. 2021 Feb 18;21:125. doi: 10.1186/s12935-021-01802-5 (PMC7893763; doi:10.1186/s12935-021-01802-5)
Supplement: Supplementary file 7 — Additional file 7: Table S6.Preliminary screening of CLU, APOA4, APOE and MLH3 using ELISA. [file 12935_2021_1802_MOESM7_ESM.docx]

## Additional file 7: Table S6. Preliminary screening of CLU, APOA4, APOE and MLH3 using ELISA

| Protein | ROC area | Standard error | 95% Confidence Intervals |
| --- | --- | --- | --- |
| CLU | 0.592 | 0.069 | 0.457, 0.728 |
| APOA4 | 0.650 | 0.063 | 0.527, 0.772 |
| APOE | 0.599 | 0.063 | 0.476, 0.772 |
| MLH3 | 0.677 | 0.058 | 0.564, 0.791 |
