# Supplementary material for: Discovery and validation of FBLN1 and ANT3 as potential biomarkers for early detection of cervical cancer
Source: Cancer Cell Int. 2021 Feb 18;21:125. doi: 10.1186/s12935-021-01802-5 (PMC7893763; doi:10.1186/s12935-021-01802-5)
Supplement: Supplementary file 12 — Additional file 12: Table S8.List of target protein and peptide sequences for PRM quantification. [file 12935_2021_1802_MOESM12_ESM.docx]

**Additional file 12: Table S8. List of target protein and peptide sequences for PRM quantification**

| **Protein name** | **Peptide sequence** | **Charge** | **m/z** |
| --- | --- | --- | --- |
| sp\|P23142\|FBLN1_HUMAN | SQETGDLDVGGLQETDK | 2 | 896.415784 |
|  | IIEVEEEQEDPYLNDR | 2 | 995.965642 |
| sp\|P02649\|APOE_HUMAN | SELEEQLTPVAEETR | 2 | 865.925788 |
|  | LAVYQAGAR | 2 | 474.766708 |
| sp\|P06727\|APOA4_HUMAN | LGEVNTYAGDLQK | 2 | 704.359356 |
|  | IDQNVEELK | 2 | 544.285128 |
| sp\|P38646\|GRP75_HUMAN | VDNALQSGNSQESVTEQDSK | 2 | 1068.488001 |
| sp\|P01008\|ANT3_HUMAN | TSDQIHFFFAK | 2 | 670.835319 |
|  | VAEGTQVLELPFK | 2 | 715.898117 |
| sp\|P02647\|APOA1_HUMAN | DLATVYVDVLK | 2 | 618.347728 |
|  | VSFLSALEEYTK | 2 | 693.8612 |
| AQUA Peptide | DSPSAPVNVT**V**R | 2 | 624.334763 |
